# Circulating leptin and its muscle gene expression in Nellore cattle with divergent feed efficiency

**DOI:** 10.1186/s40104-017-0203-3

**Published:** 2017-09-01

**Authors:** Lúcio Flávio Macedo Mota, Cristina Moreira Bonafé, Pâmela Almeida Alexandre, Miguel Henrique Santana, Francisco José Novais, Erika Toriyama, Aldrin Vieira Pires, Saulo da Luz Silva, Paulo Roberto Leme, José Bento Sterman Ferraz, Heidge Fukumasu

**Affiliations:** 1 Departmento de Zootecnia, Universidade Federal dos Vales do Jequitinhonha e Mucuri, Diamantina, MG 39100-000 Brazil; 2 Present adress: Faculdade de Ciências Agrárias e Veterinárias, Universidade Estadual Paulista, Jaboticabal, SP 14884-900 Brazil; 3 Departamento de Medicina Veterinária, Faculdade de Zootecnia e Engenharia de Alimentos, Universidade de São Paulo, Av. Duque de Caxias Norte n°225, Pirassununga, 13635-900 SP Brazil; 4 Departmento de Zootecnia e Desenvolvimento Agrossocioambiental Sustentável, Faculdade de Veterinária, Universidade Federal Fluminense, Niteroi, RJ 24230-340 Brazil; 5 Departamento de Zootecnia, Faculdade de Zootecnia e Engenharia de Alimentos, Universidade de São Paulo, Pirassununga, 13635-900 SP Brazil

**Keywords:** Beef cattle, Energy homeostasis, Fat depositon, Residual feed intake

## Abstract

**Background:**

Leptin has a strong relation to important traits in animal production, such as carcass composition, feed intake, and reproduction. It is mainly produced by adipose cells and acts predominantly in the hypothalamus. In this study, circulating leptin and its gene expression in muscle were evaluated in two groups of young Nellore bulls with divergent feed efficiency. Individual dry matter intake (DMI) and average daily gain (ADG) of 98 Nellore bulls were evaluated in feedlot for 70 d to determinate the residual feed intake (RFI) and select 20 animals for the high feed efficient (LRFI) and 20 for the low feed efficient (HRFI) groups. Blood samples were collected on d 56 and at slaughter (80 d) to determine circulating plasma leptin. Samples of *Longissimus dorsi* were taken at slaughter for leptin gene expression levels.

**Results:**

DMI and RFI were different between groups and LRFI animals showed less back fat and rump fat thickness, as well as less pelvic and kidney fat weight. Circulating leptin increased over time in all animals. Plasma leptin was greater in LRFI on 56 d and at slaughter (*P* = 0.0049). Gene expression of leptin were greater in LRFI animals (*P* = 0.0022) in accordance with the plasma levels. The animals of the LRFI group were leaner, ate less, and had more circulating leptin and its gene expression.

**Conclusion:**

These findings demonstrated that leptin plays its physiological role in young Nellore bulls, probably controlling food intake because feed efficient animals have more leptin and lower residual feed intake.

## Background

Leptin is a polypeptide hormone produced mainly by adipose cells [1], being also expressed by other tissues such as skeletal muscle [2], mammary gland [3], and others [4]. The main physiological function of leptin is energy homeostasis by controlling feed intake by inhibiting hunger [5–7]. It acts on a specific receptor (LEPR) in the brain, most specific to the hypothalamus in ventromedial, dorsomedial, and arcuate nuclei [8]. Although the physiological role of leptin is to control food intake, obese individuals generally present greater circulating levels of the hormone due to their greater percentage of body fat [9]. Obese people usually present resistance to leptin by alterations in the *LEPR* signaling and/or diminished crossing into the blood brain barrier [10].

Fat metabolism and deposition is important for livestock, affecting the quality of the products (beef, milk, etc) and providing energy reserves for reproduction and lactation [11]. Soon after the discovery of the obese gene in 1994 [1], its product, leptin, was identified for potential applications in animal production [12]. Indeed, research has been done on different aspects of leptin in livestock, including the use of its polymorphisms in animal breeding programs [13–17] and the use of circulating plasma leptin as a predictor of body composition [18, 19].

In sheep, plasma concentration of leptin is more related to variation in body fatness (35%) than to nutritional status (17%) [20]. Corroborating this result, leptin levels in beef cattle (*Bos taurus*) were positively associated with adipose cell size [21], body fatness [22], and with the 12^th^ rib and rump fat thickness, explaining 16.8% of the variation in the 12^th^ rib fat thickness [19]. In the same work, the authors [19] showed the circulating leptin was positively associated with residual feed intake (RFI), a measure of feed efficiency, however, they explained very little of the variation in RFI (<3.2% of the variance). A moderate association (*r* = 0.31) of leptin with RFI was also showed by Richardson et al. [23] in Angus steers.

In *Bos indicus*, the majority of beef cattle grown in Brazil, *Leptin* gene polymorphisms were associated mostly with growth and carcass traits [17, 24, 25], but no study was found on the circulating plasma leptin and/or muscle gene expression association with feed efficiency. Thus, the aim of this work was to characterize the circulating plasma leptin and its muscle gene expression in young Nellore bulls with divergent residual feed intake.

## Methods

### Animals and experimental design

A feeding trial of 98 Nellore bulls (16 to 20 months old and 376 ± 29 kg BW) was conducted at the Faculdade de Zootecnia e Engenharia de Alimentos, Universidade de São Paulo (FZEA / USP), Pirassununga, SP, Brazil. All the details regarding animals, traits, and diet can be found in Alexandre et al. [26]. Briefly, the data collection period consisted of 70 d preceeded by 21 d of adaptation to diet and environment. Animals were weighed in the beginning, the end, and every 14 d of the experimental period. Additionally, daily dry matter intake (DMI) for each animal was measured by weighting the orts every day. Residual feed intake (RFI) was calculated [27], and two groups were formed: low RFI (LRFI) and high RFI (HRFI), each composed of 20 extreme animals.

### Plasmatic leptin quantification

Plasma samples were collected at 56 d of feeding trial and at slaughter (80 d) by jugular venipuncture using vacutainer tubes containing sodium heparin as anticoagulant (BD Vacutainer Plus, BD, Brazil). All blood samples were centrifuged at 3,500×g for 15 min at 4 °C, and plasma was collected and stored at −20 °C until assayed for leptin. The concentration was determined in duplicate 100 μL aliquots of plasma samples, using the leptin RIA kit (Multi-species leptin RIA kit, Cat. # XL-85 K, Millipore, St. Charles, MO, USA) and following the manufacturer’s instructions. Intra and interassay CVs for the leptin assay were less than 10% as described by Delavaud et al. [20].

### Slaughter and tissue sample collection

Animals were slaughtered on d 80 at the Experimental slaughterhouse of University of São Paulo, after fasting from feed for 16 h. Immediately after slaughter, samples of the medial portion from the right *Longissimus lumborum* muscle (between the 12^th^ and 13^th^ ribs) were taken from 10 animals of each RFI group. The samples were collected and immediately immersed in RNA stabilization solution (RNAholder – Bioagency, São Paulo/SP, Brazil). The sample were maintained overnight at 4 °C and then stored at −80 °C until RNA extraction.

### Gene expression of *Leptin*

Total RNA was extracted from 300 mg of powdered tissue samples using Trizol reagent following the manufacturer’s protocol (Invitrogen, Carlsbad, CA, USA). After quantification in a spectrophotometer, 1 μg of total RNA was treated with DNAse (Invitrogen, Carlsbad, CA, USA) and reverse transcribed into cDNA using Go Script ™ Reverse Transcription System kit (Promega Corporation, Madison, WI, USA). Real time-PCR was performed on a CFX ConnectTM Real-Time PCR detection system (Bio-Rad, Hercules, CA, USA), using SYBR Green RT-PCR kit (Applied Biosystems, Foster City, CA, USA) with the following cycle parameters: 95 °C for 3 min and 40 cycles at 95 °C for 10 s and 60 °C for 30 s. Primers utilized for PCR were leptin (F) 5’GGGCACGTCAGCATCTATTA3’ and leptin (R) 5’CCTGTCTGCTGTTATGGTCTTA3’, and for the endogenous control, the ribosomal 18S (F) 5’CCTGCGGCTTAATTTGACTC3’ and 18S (R) 5’AACTAAG-AACGGCCATGCAC3’ were used. The amplification efficiency was 0.90 to 0.99 for leptin and 18S, respectively. All reactions were performed in triplicate, and the method of Livak and Schimttgen was used for gene expression analysis [28].

### Statistical analysis

All analyses were performed using GraphPad Prism version 6.0 for Mac (GraphPad Sotware, La Jolla California, USA). The phenotypic measures assessed in the LRFI and HRFI groups were first tested for Gaussian distribution by the Shapiro-Wilk test, and later, they were tested for difference between the means of the groups by Student’s t-test for normal distributed data and Mann–Whitney-Wilcoxon test for nonparametric data. The correlation between the two leptin measures was performed with the Pearson test. A two-way ANOVA followed by the Fisher post-test was used for leptin plasmatic concentration on d 56 and d 80. Statistically significant results were considered when *P* < 0.05.

## Results

### Characterization of feed efficiency groups

Phenotypic traits (related to body weight, feed efficiency, muscle and fat deposition, liver weight, and cascass yeld), are already published and no significant difference of sire or age on RFI was found [26]. Animals of LRFI and HRFI groups showed no difference in body weight (initial, final, or average daily gain) and rib eye area (initial, final, and gain). On the other hand, DMI and RFI were different between groups, wherein LRFI animals presented less DMI [26]. In addition, LRFI animals presented less fat deposition in both back fat and rump fat and less pelvic and kidney fat relative weight in comparison with HRFI. Thus, LRFI animals eat less and are leaner than HRFI animals [26].

### Circulating plasma leptin and muscle gene expression

**Fig. 1:**
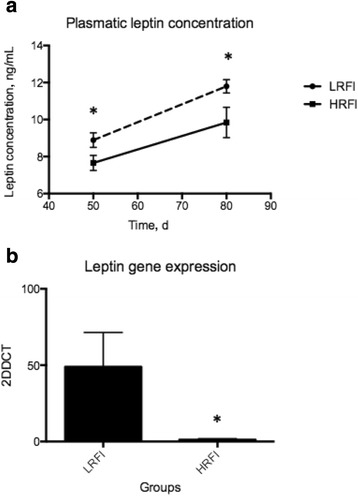
Circulating plasma leptin and muscle gene expression in young Nellore bulls divergently selected for feed efficiency. a Circulating plasma leptin were evaluated on d 50 and at the end of the experiment on d 80. b Leptin gene expression in muscle was performed at the end of the experiment on d 80. * notes statistical difference (*P* < 0.05)

The correlation between those two measures was moderate to strong and significant (*r* = 0.5817 and *P* = 0.0071). In this experiment, leptin concentration in plasma increased over time in both groups (Fig. 1a), accounting for 21.3% of the total variation and was considered significant (*P* < 0.0001). In addition, leptin concentration was different between groups (*P* = 0.0049). However, no interaction between time and groups was noted though (*P* = 0.513).

Leptin gene expression was performed in muscle samples collected at slaughter. LRFI animals showed greater expression of leptin (*P* = 0.0022) compared to HRFI animals, reflecting the increased leptin plasma concentration of animals at slaughter (Fig. 1b).

## Discussion

In this study, it was shown that circulating plasma leptin and muscle gene expression were greater in high feed efficiency group (LRFI) than in low feed efficiency group (HRFI) of Nellore bulls. In addition, feed efficient animals showed less fat deposition, probably a consequence of significant less DMI. In a previous work from the group, it was demonstrated that LRFI animals have less circulating plasma cholesterol at the end of the feeding trial [26]. Since the main role of leptin is regulate food intake in different species [29–31], these results support the physiological role of this hormone in Nellore cattle, since leptin is controlling DMI, plasma cholesterol, and fat deposition. Supporting this concept, less efficient animals have less muscle gene expression and circulating leptin which increases DMI, plasmatic cholesterol, and fat deposition.

Nonetheless, in other experiments on cattle, leptin concentration was positively associated with DMI, body fat, and RFI ([19, 23, 32]. In these cases, leptin seems to be related to body fat mass but not with the control of food intake. Interestingly, this phenotype resembles the resistance to leptin in obese humans where even elevated levels of leptin produced by greater percentage of body fat failed to control hunger and modulated body fat [33]. In accordance with the hypothesis, a recently published work from Foote et al. [34] suggested that beef cattle on a high grain finishing ration could become slightly leptin resistant.

The opposing results observed in the current study and others suggest the hypothesis that animals in this experiment are not in an “obese” phenotype, being instead where leptin reflects fat body mass. This could be explained by the conjunction of: (1) having used young bulls while others used steers [18, 19, 23, 34] or heifers [34, 35] and/or; (2) Nellore is an indicine breed and most experiments on cattle are performed in taurine breeds. The first possibility is probably the most relevant since the effect of sex on fat deposition has long been described. Total rate of fat deposition relative to muscle is similar for heifers and steers but significantly lower in bulls [36]. These authors concluded that differences in fattening patterns among sexes result from a combination of fattening at a lighter weight of carcass muscle in heifers than steers and in steers than bulls, in addition to a more rapid rate of fat deposition relative to muscle in heifersand steers compared to bulls. In addition, the effect of sex is well known to be related to feed efficiency, where bulls are more efficient than steers or heifers [37, 38]. In humans, females have greater circulating leptin as compared to males, even after the correction for differences in body fat mass [39]. In vitro experiments with human adypocytes in primary culture showed that both testosterone, and its biologically active metabolite dihydrotestosterone inhibited leptin secretion up to 62%, supporting that sex is clearly a relevant factor on leptin metabolism [39].

One should also consider the possible effect of genetic differences between Nellore and taurine breeds for the observed effect in this experiment. Indicine cattle are known to be more adapted to tropical climate but with less growth performance than taurine breeds. Corroborating this idea, Marcondes et al. [40] demonstrated the dry matter intake and performance of steers were higher in Nellore crossbreds (Nellore-Simmental and Nellore-Red Angus) than that of pure Nellore, however, no difference in feed efficiency was noted between groups. Paschal and colleagues [41] studying the postweaning and feedlot growth and carcass characteristics of five indicine breeds and one taurine (Angus) showed that Zebu crosses (including Nellore) grew faster postweaning and were heavier and taller than Angus crosses. However, the Angus cross was more desirable in marbling score and quality grade. Beef products derived from Nellore are recognized by the international market as very lean meat due to lack of intramuscular fat [42]. The same authors demonstrated the difference in intramuscular fat content in skeletal muscle of Nellore and Angus cattle is due to a slightly enhanced muscle adipogenesis in Angus cattle. Altogether, these results support the concept that Nellore have diferent growth curves in comparison to taurine breeds taking more time for fat deposition.

Therefore, it is plausible that the increased muscle gene expression of leptin and circulating leptin levels in feed efficient young Nellore bulls in comparison to less efficient animals, although, others demonstrated the opposite. This result also calls attention for the use of leptin as a possible predictor for body fat mass in cattle, especially if considered for use in Nellore cattle.

## Conclusion

To the best of our knowledge, this work demonstrated for the first time that leptin plays its physiological role in young Nellore bulls, rather its pathological role, controlling food intake because feed efficient animals have more circulating leptin and lower residual feed intake.

## Abbreviations

ADG: Average daily gain
BWf: Final body weight
BWg: Body weight gain
BWi: Initial body weight
DMI: Dry matter intake
HRFI: Low feed efficient
RBg: Rib eye gain
REAf: Final rib eye area
REAi: Initial rib eye area
RFI: Residual feed intake
RIA: Radioimmunoassay
LEPR: Leptin receptor
LRFI: High feed efficient
